# Metabolic Physiology of the Invasive Clam, *Potamocorbula amurensis:* The Interactive Role of Temperature, Salinity, and Food Availability

**DOI:** 10.1371/journal.pone.0091064

**Published:** 2014-03-05

**Authors:** Nathan A. Miller, Xi Chen, Jonathon H. Stillman

**Affiliations:** 1 Romberg Tiburon Center and Department of Biology, San Francisco State University, Tiburon, California, United States of America; 2 Department of Integrative Biology, University of California, Berkeley, California, United States of America; The Evergreen State College, United States of America

## Abstract

In biological systems energy serves as the ultimate commodity, often determining species distributions, abundances, and interactions including the potential impact of invasive species on native communities. The Asian clam *Potamocorbula amurensis* invaded the San Francisco Estuary (SFE) in 1986 and is implicated in the decline of native fish species through resource competition. Using a combined laboratory/field study we examined how energy expenditure in this clam is influenced by salinity, temperature and food availability. Measures of metabolism were made at whole organism (metabolic rate) and biochemical (pyruvate kinase (PK) and citrate synthase (CS) enzyme activities) levels. We found in the field, over the course of a year, the ratio of PK to CS was typically 1.0 suggesting that aerobic and fermentative metabolism were roughly equivalent, except for particular periods characterized by low salinity, higher temperatures, and intermediate food availabilities. In a 30-day laboratory acclimation experiment, however, neither metabolic rate nor PK:CS ratio was consistently influenced by the same variables, though the potential for fermentative pathways did predominate. We conclude that in field collected animals, the addition of biochemical measures of energetic state provide little additional information to the previously measured whole organism metabolic rate. In addition, much of the variation in the laboratory remained unexplained and additional variables, including reproductive stage or body condition may influence laboratory-based results. Further study of adult clams must consider the role of organismal condition, especially reproductive state, in comparisons of laboratory experiments and field observations.

## Introduction

Within biological communities energy serves as the ultimate commodity, determining broad patterns of distribution, abundance, persistence, and migration [1], [2]. Accurately predicting changes in such ecological patterns requires a fundamental understanding of those factors that drive energy availability and an organism’s energy demands [3]. Identifying how a species, especially an invasive species, will impact a native habitat, its potential to expand its range, and its persistence in a changing environment all require an understanding of how that species processes and exchanges energy with the environment [4], [5] (e.g., through feeding mode and intensity).

Filter feeding by an introduced clam, *Potamocorbula amurensis*, that invaded the San Francisco Estuary (SFE) in 1986 [6] is thought to be the cause of a dramatic reduction in summertime phytoplankton abundance in the shallow brackish northern parts of the SFE [7]. Historically, summer phytoplankton blooms were common in the SFE, but since the arrival of *P. amurensis*, phytoplankton abundance has been consistently low and chlorophyll levels in the estuary are typically ∼3−4 µg l^−1^ [8]. In addition to phytoplankton, *P. amurensis* is capable of grazing on bacterioplankton [9], copepod nauplii [10], and microzooplankton [11], and thus is able to graze on the entire bottom of the food web. A decline in abundance of three common estuarine copepod species coincided with the invasion of *P. amurensis* [10], probably through a combination of predation and competition for phytoplankton and microzooplankton resources. Concurrent declines in the abundance of higher trophic level pelagic taxa including delta smelt *Hypomesus transpacificus* [12], mysid shrimp *Neomysis mercedis* [13], longfin smelt *Spirinchus thaleichthys* [14], and striped bass *Morone saxatilis* [15] have occurred, all presumably due to food limitation.

The distribution of *P. amurensis,* and therefore its immediate impact, has been assumed to be limited by energetic constraints associated with responding to and tolerating low salinity stress [16], [17], [18]. However, in previous work we have demonstrated that energy expenditure (as metabolic rate) was uncorrelated with salinity, as well as, temperature and chlorophyll a concentration, calling this assumption into question [19]. However, estuarine systems are dynamic, with environmental variables varying on hourly, seasonally, and even decadal cycles. Consequently, a single environmental measurement may not accurately capture the complexity of the salinity/temperature/chlorophyll field a given clam may experience.

Metabolic rates represent an integrative, overall measure of energetics with the potential for a more nuanced assessment using biochemical indicators [20], [21], [22]. Additionally, the fact that bivalves can potentially isolate their tissues from unfavorable environmental conditions through valve closure may mask some of the direct effects of environmental conditions, though to date it is unclear whether *P. amurensis* utilizes such isolation behavior. If so, such isolation could result in the use of fermentative metabolism, which can be characterized by upregulation of fermentative biochemical pathways.

We combined a biochemical assessment of metabolic state from field collected clams with a factorial laboratory experiment in which we manipulated salinity, temperature, and food availability. We sought to determine if clam metabolic physiology is, in fact, unrelated to these environmental variables under controlled conditions and when viewed from a biochemical standpoint (measuring pyruvate kinase and citrate synthase activities). Pyruvate kinase, a glycolytic enzyme, provides a measure of lower efficiency fermentative metabolism and citrate synthase, a tricarboxylic acid (TCA) cycle enzyme providing a measure of higher efficiency, aerobic metabolism. The relative predominance of these two enzymes, therefore, provides insights into the relative importance of aerobic versus fermentative metabolic pathways [23]. Here we report whether biochemical assays can be useful indicators of changes in *P. amurensis* metabolic state across environmental gradients in temperature, salinity and food availability in field and controlled laboratory conditions.

## Materials and Methods

### Ethics Statement

This study did not involve endangered or protected species and specific permissions were not required for activities at locations.

### Field Sampling

From November 2010 to October 2011, *P. amurensis* were collected from a shoal area in Suisun Bay (38° 3.6′N, 122° 2.1′W, ∼65 river km) on a roughly monthly schedule as part of a larger project that included measurement of clam respiration rates immediately upon collection [19]. Clams were collected by Van Veen grab from the RV Questuary, separated from the sediment, immediately frozen on dry ice, and stored at −80°C in preparation for metabolic enzyme analysis. Water samples were collected approximately 0.5 m above the sediment surface using a Seabird carousel water sampler with an attached CTD (recording bottom salinity and temperature). Chlorophyll concentration was determined following the methods outlined in [21]. Salinity is expressed on the Practical Salinity Scale (PSS) and therefore is reported without units.

### Laboratory Experimental Setup

*P. amurensis* for the laboratory experiment were collected by Van Veen grab from the RV Questuary on July 12, 2012 from a shallow shoal area in Suisun Bay (38° 3.6′N, 122° 2.1′W, ∼65 river km). They were held in 9L plastic tubs containing 3 cm of crushed aragonite aquarium sand, at 10 salinity and 15°C, and fed three times per week with 1∶5 diluted Shellfish Diet 1800 (Reed Mariculture) for 3 weeks. Temperature and salinity records for the three months prior to collection were obtained from US Bureau of Reclamation sensors near our collection site (http://cdec.water.ca.gov/cgi-progs/stationInfo?station_id=PCT) to examine the clams’ prior acclimatization history.

Following the initial laboratory acclimation, clams were used in a 30-day acclimation experiment with a fully-factorial, three factor design with three levels of salinity (2, 8, and 15), two levels of temperature (12 and 18°C), and two levels of food (LOW: 1x per week of 3 mL of 1∶5 diluted Shellfish Diet, or HIGH: 5x per week of 3 mL of 1∶5 diluted Shellfish Diet). Clams of equivalent sizes (mean +/−95% confidence interval, length: 15.5+/−0.4 mm, width: 10.1+/−0.2 mm, height: 6.2+/−0.2) were randomly assigned to one of n = 72 600 mL beakers, each containing 150 g of clean, crushed aragonite sand (Aragamax, Brand Carib Sea). Each beaker was randomly assigned a food, salinity and temperature level resulting in a total of six replicate beakers per treatment, 48 clams per treatment combination, 576 clams total. Brackish water (salinity 2, 8, and 15) was created by mixing artificial fresh water [24] with a commercially available, artificial sea salt mix (AquaVitro Salinity). Each beaker was filled to 500 mL with the appropriate treatment water. Large batches of water at each test salinity were mixed at the beginning of the experiment so that the same batch could be used throughout the entire experiment. Temperatures were controlled by immersing and randomly assorting the beakers in two temperature controlled sea tables held at 12±1 and 18±1°C by chillers and immersion heaters. The two food levels were then randomly assigned to each beaker. Each beaker also contained a small bubbler and a loosely fitting plastic petri dish lid to minimize evaporation.

The experiment was run for 30 days, with weekly changes of ∼2/3 of the water in each beaker. At that time clam survival was also assessed. The acclimation duration was chosen to provide the greatest potential for metabolic physiology to respond to differential food availability [25], as well as, provide insights into how these clams respond to longer-term, sub-lethal environmental stress. At the end of the experiment 10 clams were randomly removed from each of the 12 treatments to measure oxygen consumption rates (see below). The remaining clams were removed, flash-frozen in liquid nitrogen, and stored at −80°C for subsequent analysis of metabolic enzyme activity.

### Metabolic Enzyme Activity

The activity of pyruvate kinase (PK) and citrate synthase (CS) was measured on six clams from each field sampling date and six clams from each laboratory treatment. From each clam, mantle and foot tissue were dissected, weighed, and placed in individual 1.5 mL tubes. Each sample was individually homogenized in a 30-fold dilution of homogenization buffer (50 mM K_3_PO_4_, pH 6.8 at 20°C) by brief sonication on ice. Samples were then centrifuged at 16,100×**g** for 15 min at 4°C. Following centrifugation supernatants were transferred to new tubes, briefly mixed, and centrifuged at 16,100×**g** for 10 sec. Homogenates were stored on ice.

### Citrate Synthase Activity

Citrate synthase activity was measured following methods in [26], modified for a microplate analysis. An initial 5 µL of homogenate (mantle and gill from each clam) was added to the wells of a clear-bottomed, black-walled, 96-well microplate. For each clam/tissue combination, six wells were designated OAA+ and six were designated OAA−, with the six wells serving as technical replicates (repeated analysis of the same homogenate). The OAA+ wells received 202.5 µL of CS+ cocktail (42.5 mM Imidizole/HCl, 1.5 mM MgCl2, 0.1 mM DTNB/Ellman’s Reagent, 12.4 µM Acetyl-CoA, 0.25 mM oxaloacetic acid (OAA)). The OAA− wells received the same volume of CS- cocktail in which the OAA was replaced with the same volume of Imidizole/HCl buffer. Both cocktails were stored on ice prior to use. The cocktail solution was added to the wells using a multi-channel pipette and the change in absorbance (at a wavelength of 415 nm) in each well over time was measured at 20°C. Measurements were made every 5 s for a total of 2 min. In this assay the measured absorbance changes even when no OAA is added to the reaction. To account for this background change we subtracted the rate change of OAA− from the rate change of OAA+ wells to obtain an accurate measure of CS activity. The CS activity (µmoles oxaloacetate reacted per min) was expressed as IU per milligram sample tissue.

### Pyruvate Kinase Activity

The methods of [26] were modified for microplate measurements of pyruvate kinase activity. An initial 5 µL of each homogenate was added to each of 6 wells of a clear-bottomed, black-walled, 96-well microplate, followed by 200 µL PK assay cocktail: 160 mM Tris/Cl, 200 mM KCl, 20 mM MgSO4, 200 µM fructose bisphospate along with 1.0 mM PEP, 5 mM ADP, 150 µM NADH, and 50 Units/mL LDH. Following the addition of the cocktail, the change in absorbance in each well (at a wavelength of 340 nm) was measured every 5 s for a total of 2 min at 25°C. The PK activity (µmoles pyruvate generated per min) was expressed as IU per milligram sample tissue. Both enzyme activity assays were performed using a Tecan Infinite F200 microplate reader.

### Oxygen Consumption Rates

At the conclusion of the experiment, oxygen consumption rates of ten clams from each treatment were measured as a proxy for metabolic rate. To ensure that all clams were measured at the same period post-feeding all the clams from the “low food” treatments (60 clams) were measured on the first day (1 day following their weekly feeding) and all clams from the “high food” treatments were measured the following day (1 day following their most recent feeding). To measure oxygen consumption rates, 10 clams were randomly chosen from each treatment and transferred to 30 mL glass, scintillation vials containing 16 g of clean aragonite sand and the sterile filtered water of the appropriate salinity. Planar optode spots (diameter 0.5 cm; Presens, Germany) had been previously secured to the inside of each vial and calibrated in the laboratory using O_2_-saturated and O_2_-free seawater water. Each vial was filled with water and sealed with a screw lid ensuring no bubbles were trapped within the vial. The cap of each vial was fitted with a magnetic “propeller” so that when placed on a laboratory stir-plate the propeller slowly turned, ensuring the water within the vial was well mixed. The vials were submersed in a temperature controlled water bath (ThermoScientific RTE7) held at 15°C for all measurements. Metabolic rate measurements were conducted at a single common temperature of 15°C to determine how a 30-day acclimation to various temperatures, salinities, and food levels might alter clam metabolism. The use of a common temperature stemmed from the fact that if measurements were made on individuals at their acclimation temperatures (12 or 18°C) differences in metabolic rate would be apparent simply due to the fact that the measurements were made at different temperatures (representing a Q_10_ effect). To detect the potential for structural or biochemical changes that might occur during acclimation and consequently influence energy metabolism, our measurements were made at a common intermediate temperature (15°C). In this way, the Q_10_ effects associated with different measurement temperatures did not confound differences in metabolic rate.

Every 10 min individual vials were removed from the water bath, placed in a temperature controlled aluminum block atop a laboratory stir-plate, and oxygen saturation within the vial measured with a Fibox3 mini oxygen sensor (Presens, Germany). The oxygen levels in each vial were never allowed to drop below 70% air saturation. After 70 min, clams were removed from the vials, dissected from their shells, weighed, dried at 60°C for 24 h and reweighed to determine both wet and dry tissue mass. Oxygen consumption rates were reported as µmole O_2_ consumed per gram dry mass per hr.

### Statistical Analyses

All statistical analyses were carried out using the R statistical program [27]. Main effects and interaction effects of temperature, salinity, and food level on clam metabolic rates and PK:CS ratios were tested using multiple linear regression models. Initial models were considered full and contained all terms and interactions. During model selection the most complex term(s) were removed sequentially and the model rerun. The process was repeated until removal of an additional term resulted in a poorer model fit than the prior model (following [28]). Regression model comparisons were made using the *AIC* function in the *base* package. Models with lower AIC values were considered to better fitting and residual errors were tested for normality and homogeneity using visual assessment of QQ normality plots and residual boxplots. Pearson correlations were calculated using the *cor.test* function in the *base* package. All mentions of ‘significance’ are in reference to the results of a particular statistical test using α of 0.05 unless otherwise stated.

## Results

### Field Collected Clams

**Figure 1:**
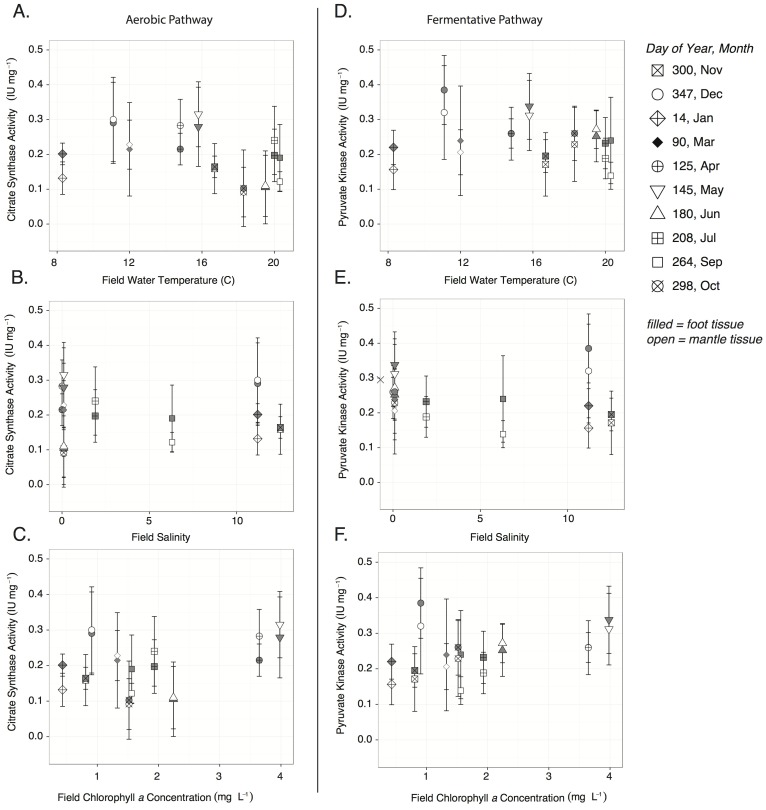
Enzyme activities in field collected clams. Citrate synthase (**A, B, C**) and pyruvate kinase (**D, E, F**) activity (IU mg wet tissue^−1^) in clam mantle and foot tissue, as a function of field temperature (°C), salinity, and chlorophyll *a* concentration (mg L^−1^) for field sampling dates from Nov 2010-Oct 2011. Citrate synthase represents the potential for aerobic metabolic pathways, while times when pyruvate kinase activity exceeds that of citrate synthase represent the potential for fermentative pathways to predominate. Values represent means, n = 6.

**Figure 2:**
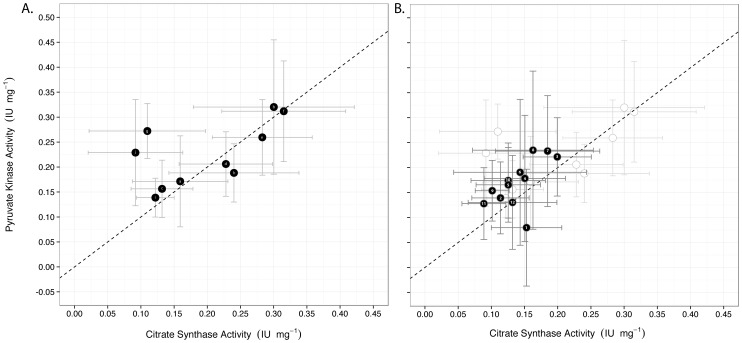
Relationship between citrate synthase and pyruvate kinase activity (IU mg wet tissue^−1^) in mantle tissues in A field collected clams and B clams from laboratory experiment. Values represent means +/− SE, n = 6 for each point. In **A,** letters within the points identify each environmental variable combinations at the time of clam collection (temperature, °C | salinity | chlorophyll a concentration, µg L^−1^), a) 16.7 | 12.5 | 0.8, b) 11.1 | 11.2 | 0.9, c) 8.3 | 1.2 | 0.4, d) 12 | 0.1 | 1.3, e) 14.8 | 0.0 | 3.6, f) 15.8 | 0.1 | 4.0, g) 19.5 | 0.1 | 2.2, h) 20 | 1.9 | 1.9, i) 20.3 | 6.3 | 1.6, j) 18.3 | 0.1 | 1.5. In **B**, numbers within the points identify each treatment combination (salinity | temperature, °C | food level). 1) 2 | 12 | High, 2) 2 | 12 | Low, 3) 2 | 18 | High, 4) 2 | 18 | Low, 5) 8 | 12 | High, 6) 8 | 12 | Low, 7) 8 | 18 | High, 8) 8 | 18 | Low, 9) 15 | 12 | High, 10) 15 | 12 | Low, 11) 15 | 18 | High, 12) 15 | 18 | Low. Unfilled points in the background of **B** represent data from field clams (shown in **A**) and are provided for comparison.

Enzyme activities in field-collected clams were not strongly correlated with temperature, salinity, or chlorophyll a concentration (Figure 1). Citrate synthase activity was routinely greater in mantle than foot tissue (mantle: 0.14±0.007, foot: 0.133±0.007, mean ± SE) and pyruvate kinase activity routinely greater in foot than mantle tissue (foot: 0.16±0.009, mantle: 0.169±0.012) (Figure 1). The two enzymes were strongly correlated in mantle tissue (Figure 2) (Pearson correlation = 0.58, t_58_ = 5.47, p<0.001), though on two dates (DOY: 180 and 298) PK activity exceeded CS activity (the same pattern was found in foot tissue, *not shown*). If these two dates are removed the correlation between PK and CS is 0.87 illustrating that these points are considerably different from the others. These dates are associated with relatively warm water temperatures (19.5 and 18.3°C), relatively low salinities (both 0.1), and low to intermediate chlorophyll levels (2.2 and 1.5 µg L^−1^) (Figure 2A). Under these conditions the potential for fermentative metabolism (PK) appears to predominate over aerobic metabolism (CS).

### Laboratory Acclimation Experiment

**Figure 3:**
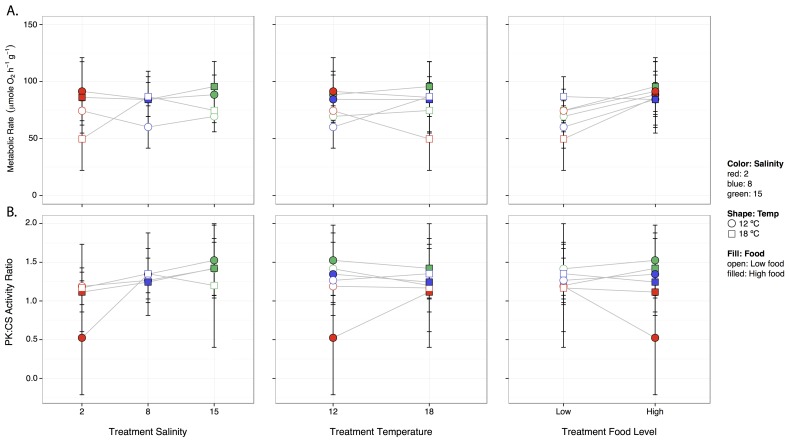
Laboratory measurements of clam metabolism under each treatment condition. Metabolic rate **A** and PK:CS ratio **B** as a function of treatment temperature (panel 1), treatment salinity (panel 2), and treatment food level panel 3, in the laboratory. Values represent means +/−95% confidence intervals, n = 10 in A and n = 6 in B.

In the laboratory, clam survival was slightly lower in the high salinity, high temperature treatments (72.5 and 75% survival, Figure S1), but there were no strong patterns between the treatments of salinity, temperature, or food level on metabolic rates. Variance in metabolism was smaller at higher food levels (Figure 3A, panel 3), but there was no other clear pattern with temperature or salinity (Figure 3A, panel 1 and 2). The pattern was reversed for PK:CS ratio where low food groups had the smallest variation in PK:CS ratio (Figure 3B, panel 3). Again, there were no other strong patterns of temperature, salinity, or food level on PK:CS ratio. The best linear regression model (based on AIC comparisons) explaining variation in clam metabolic rates included a two-way interaction between temperature and salinity, as well as main effects of temperature, salinity, and food (Table 1A, Adjusted R^2^∶0.1). However, despite the importance of these terms (based on AIC values for models with and without them), only the main effect of food was statistically significant (Linear regression: F_1,109_ = 13.32, p<0.001).

**Table 1:** Results of multiple linear regressions to determine the effect of experiment, temperature, salinity, and food level on **A.** metabolic rate (model: µmole O_2_ hr^−1^ g^−1^ = T+S+FL+(T * S) ), adj. R^2^ = 0.1, **B.** and PK:CS ratio (model: PK:CS ratio = S+FL+(S * FL) ), adj. R^2^ = 0.12.

A. Metabolic Rate					
	df	SS	Mean SE	F-value	p-value
Temperature (T)	1	64	63.6	0.075	0.78
Salinity (S)	2	919	459.5	0.543	0.58
Food Level (FL)	1	11278	11278	13.32	<0.001
T * S	2	4051	2025.3	2.392	0.096
Residuals	109	92289	846.7		
**B. PK:CS (mantle)**					
	**df**	**SS**	**Mean SE**	**F-value**	**p-value**
Salinity (S)	2	2.023	1.011	5.13	<0.01
Food Level (FL)	1	0.086	0.086	0.44	0.51
S * FL	2	0.855	0.427	2.17	0.12
Residuals	66	13.01	0.197		

Mantle CS and PK activities in the 30-day acclimation experiment are strongly correlated (Figure 2B) (Pearson correlation: 0.69, t_df = 70_ = 7.94, p<0.001). PK activity exceeded CS activity in most treatments except for treatment 1 (salinity: 2, temperature: 12, food level: High). The best linear regression model (lowest AIC) explaining the PK:CS ratio included a two-way interaction between salinity and food (Table 1B) and the main effects of salinity and food. The interaction was non-significant (Linear regression: F_2,66_ = 2.17, p = 0.12) though there was a main effect of salinity (Linear regression: F_2,66_ = 5.13, p<0.01 and the adjusted R^2^ for the model was 0.12.

## Discussion

Accurately predicting the impacts of invasive species (such as the clam, *Potamocorbula amurensis*) on the trophic transfer of energy through a food web requires an understanding of how variation in environmental parameters influences each species’ metabolic demands. We found that in field collected *P. amurensis* the ratio of PK to CS activity was routinely 1∶1 though the absolute activity varied among sampling dates. In the few studies that have considered aerobic vs. fermentative capacity in mollusks using the ratio of PK to CS, the ratio has been ∼2.0 [29], [30]. This perhaps indicates that *P. amurensis* has a greater scope for aerobic energy production than some other clams, a finding previously suggested in this species [31].

An examination of the corresponding environmental parameters in the field provides a general pattern that is suggestive of a correlation between increasing enzyme activity and increasing temperature, though higher enzyme activities at higher food levels is also a potential explanation. Two sampling dates (DOY 180 and 298) are of particular interest as the PK activity exceeded the CS activity, suggesting the potential for fermentative processes to exceed aerobic ones. These points both represent periods with relatively high water temperature (19.6 and 18°C), low salinities (both 0.0) and relatively low levels of chlorophyll *a* (2.2 and 1.5 µg/L). We hypothesize that under relatively warm conditions, when salinities are low and food is not abundant these clams utilize fermentative processes either as a result of valve closure (to avoid the low salinity) or because energy expenditures exceed the aerobic capacity to process energy at high temperature and low food. To test this hypothesis we undertook a series of laboratory experiments to clarify how temperature, salinity, and food availability influence rates oxygen consumption and the activities of metabolic enzymes.

The results of the 30-day acclimation experiment present a different story as salinity and temperature appear to have little influence upon metabolic rates, though animals at 15 salinity and 18°C did exhibit slightly reduced survival. The high food groups did have higher metabolic rates at 12°C (Figure 3A, panel 2) and across all other treatments the variability in metabolic rate was lowest in the high food group (Figure 3B, panel 3). However, we did not observe strong or consistent influences of temperature, salinity or food availability on clam metabolism such as those reported previously in field collected animals [19].

Whereas in the field high temperature, low salinity, and low food levels seemed to result in greater potential for anaerobic metabolism (greater PK:CS ratio), this was not the case in the laboratory. In addition, though the two extreme points in the field still exceeded those observed in the laboratory they no longer appear as extreme. In general, PK:CS ratios were similar across treatments (roughly 1.0–1.5), though at salinity 2, 12°C, and high food, the PK:CS ratio was roughly 0.5, suggesting the potential for greater aerobic metabolism under these conditions (Figure 3B). Such combinations are also associated with the highest metabolic rates (Figure 3A). Why this particular combination of environmental variable might elicit a greater potential for aerobic metabolism is unknown. That lack on consistency between the laboratory and field results demonstrate that while there are particular events which result in dramatic effects on energy metabolism in the field (DOY 180 and 298), these events may be rare, and elucidating what drives these responses will necessitate further study.

It is also likely that reproductive state and associated body condition influenced clam responses to salinity, temperature, and food level [32], [33,]. Clams in the field may have been in different reproductive stages, while those in the laboratory experiment (collected in July) were likely to have recently spawned [34]. As we did not assess the reproductive state or the body condition of the clams, at this time we can only speculate regarding the role of reproductive state may have played in our results. It is clear that adult *P. amurensis* show considerable variability with regards to how they respond to environmental conditions and that efforts to understand the population biology of *P. amurensis* in response to environmental conditions in the laboratory must carefully consider the timing of animal collections, as reproductive state or body condition may play as significant a role in modulating the response of energy metabolism to environmental variables.

## Supporting Information

Figure S1 **Percent clam survival in each experimental treatment at the conclusion of the 30-day laboratory acclimation experiment. Each experimental treatment began with 40 individuals.**
(TIF)
