# Supplementary figures and images for: Metabolic Physiology of the Invasive Clam, Potamocorbula amurensis: The Interactive Role of Temperature, Salinity, and Food Availability
Source: PLoS One. 2014 Mar 5;9(3):e91064. doi: 10.1371/journal.pone.0091064 (PMC3944785; doi:10.1371/journal.pone.0091064)

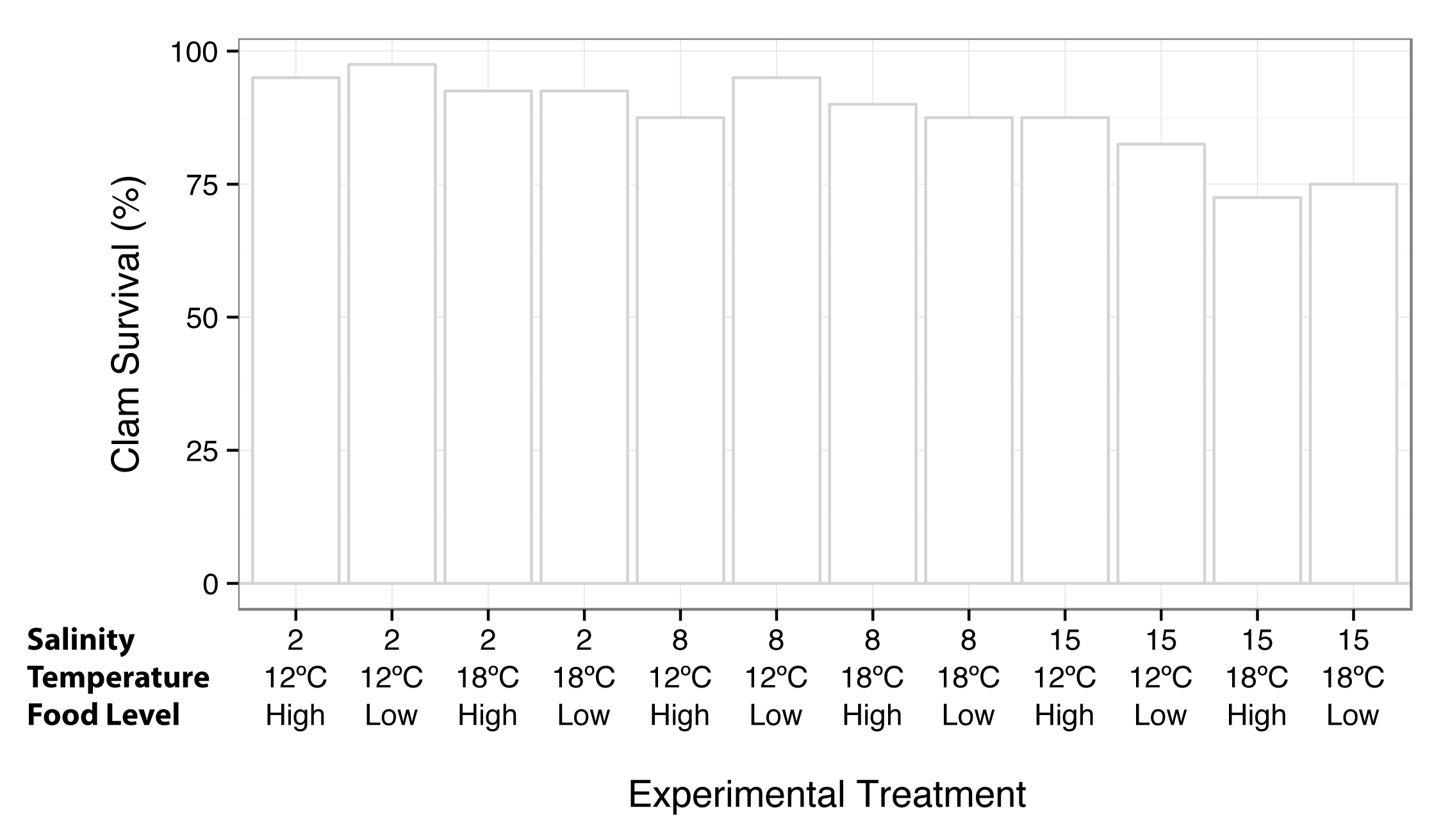

Supplement: Figure S1 — Percent clam survival in each experimental treatment at the conclusion of the 30-day laboratory acclimation experiment. Each experimental treatment began with 40 individuals. (TIF) [file pone.0091064.s001.tif]
